# Research on the Mechanism of Interaction between Styrene–Butadiene–Styrene (SBS) and Asphalt Based on Molecular Vibration Frequency

**DOI:** 10.3390/ma14020358

**Published:** 2021-01-13

**Authors:** Yuqin Zeng, Qicheng Liu, Qing Zeng, Yuhao He, Zhenghong Xu

**Affiliations:** 1 School of Physics and Electronic Science, Changsha University of Science and Technology, Changsha 410114, China; Zengyq1996@hotmail.com (Y.Z.); Quietlyzeal@126.com (Q.Z.); 2 Hunan Provincial Key Laboratory of Flexible Electronic Materials Genome Engineering, Changsha University of Science and Technology, Changsha 410114, China; 3 College of Materials Science and Engineering, Changsha University of Science and Technology, Changsha 410114, China; heyuhao_csust@163.com (Y.H.); 13955427279@163.com (Z.X.)

**Keywords:** asphalt, SBS, density functional theory, vibration spectrum, binding energy

## Abstract

Based on the four-component theory of asphalt, molecular models of the saturate, aromatic, resin, and asphaltene were constructed, respectively. The styrene–butadiene–styrene (SBS) polymer was used as the modifier. Using density functional theory (DFT) to study the effect of SBS on the molecular vibration of each component of asphalt, the vibration spectrums and binding energy of the systems composed of SBS and each component molecule of asphalt were calculated. Prepared SBS modified asphalt and measured Fourier transform infrared spectroscopy (FTIR) before and after the experiment. The results show that after SBS was added to asphalt, no chemical reaction occurred, and the system was mainly physical blending. The vibrational peak intensity of SBS and the light components of asphalt (saturate and aromatic) is stronger than that of SBS and the heavy components of asphalt (resin and asphaltene). The interaction strengths of asphalt components and polybutadiene (PB) blocks, polystyrene (PS) blocks of SBS are different. The binding energy of SBS and the saturate is the lowest and the bonding of the system is weakest. The bonding of the systems of SBS and the aromatic, resin, asphaltene is stable, and the stability of these systems are all stronger than that of SBS and the saturate.

## 1. Introduction

Asphalt is an excellent engineering material, widely used in road pavement, waterproof coating, and other fields [1,2]. Due to the nature of asphalt itself, its further application is affected by some performance defects, such as aging at high temperature and cracking at low temperature. By incorporating modifiers, modifying asphalt is the main way to solve its limited application. At present, the most widely used asphalt modifiers are polymer asphalt modifiers, mainly including styrene–butadiene–styrene (SBS), styrene–butadiene rubber (SBR), ethylene–vinyl acetate (EVA), and so on [3]. SBS is the most commonly used polymer asphalt modifier [4,5], the polystyrene end-blocks impact the strength to the polymer while the polybutadiene mid-blocks give the material its better elasticity [6]. SBS modified asphalt has good thermal stability [7], but it is very susceptible to ultraviolet (UV) radiation [8,9]. In addition, SBS modified asphalt also has good crack resistance [7], can improve the adhesion between asphalt and aggregates [10], and effectively improve the performance of asphalt.

Since SBS modified asphalt is the most widely used modified asphalt, the mechanism of interaction between SBS and asphalt has naturally been a hot research topic in the field of asphalt science. Airey [11] modified two base asphalts (A and B) with SBS and found that the extent of polymer modification has differed depending on the nature of the base asphalt and subsequently the compatibility of the asphalt-polymer system. The asphalt A showed a greater degree of polymer modification compared to the asphalt B, which is related to the larger proportion of the aromatic hydrocarbon present in the asphalt A. The content of the aromatic hydrocarbon in asphalt played an important role in compatibility between the asphalt and polymer [12]. By building models and simulating calculations, we can further observe and analyze at the microscopic molecular level, which is helpful to better understand the macroscopic experimental phenomena. Some researchers [13] used infrared spectroscopy to find that an increase in hydroxyl groups and the formation of carbonyl compounds and sulfoxides after the aging of SBS modified asphalt. The content of the aromatic hydrocarbon decreased, while the content of the resin and asphaltene increased, resulting in hardening of the sample [14]. However, the infrared spectroscopy is easily affected by the environment and equipment, while the simulation calculations are not subject to these constraints. They cover a wide range and can be studied at the microscopic molecular level. Ding et al. [15] used molecular dynamics (MD) to study the molecular agglomeration behavior of SBS and asphalt, and found that the molecular agglomeration of asphalt with longer asphaltene alkane side branches is more regular. The agglomeration structure of the asphalt molecule is more susceptible to SBS when the asphaltene alkane side branches are longer. Most of the above studies used experimental data combined with microstructure characterization to explain their modification mechanism, which is limited by the experiment and characterization equipment. At the same time, because asphalt is an extremely complex mixture, some research results cannot be effectively verified. It is even more difficult to make research at the molecular level. Currently, there are relatively few researches involving asphalt simulation calculations, and they all use MD method of the first-principles. The DFT method of the first-principles is a powerful alternative to conventional theoretical research and molecular simulation and has become a regular tool used by many researchers in chemistry, physics, chemical engineering, materials science, and other disciplines. DMol3 of Materials Studio software using DFT method to study the molecular interfacial properties, electronic structure and energetics, a board range of systems can be studied, including inorganic, organic, crystal, metal, solid, covalent solids, and infinite surfaces of a material, also can predict structure, reaction barriers, reaction energies, thermodynamic properties, and vibration spectrum [16]. It provides another effective method to study SBS modified asphalt. Moreover, the analysis and simulation of the molecular structure at the microscopic level are helpful to better understand the macroscopic experimental phenomena.

In this paper, the method of the DFT was used to observe the interaction between modifier and asphalt from a microscopic molecular scale. In order to solve the problem of difficult research on asphalt as a complex mixing system, asphalt was divided into the saturate, aromatic, resin and asphaltene. The models of the saturate, aromatic, resin, asphaltene and linear SBS with an S/B ratio of 4:4 were built. The mechanism of action between SBS and different components was studied separately. Then, SBS modified asphalt was prepared, the FTIR of SBS, asphalt, and SBS modified asphalt composite before and after the experiment were measured, and the experimental data were comprehensively analyzed. From the perspectives of molecular vibration spectrum and the binding energy between the SBS and each component of asphalt, the modification mechanism of asphalt modified by SBS at the molecular level was discussed, to some extent to make up for the deficiencies of existing researches.

## 2. Experimental

### 2.1. Calculation Models and Methods

#### 2.1.1. Calculation Models

**Figure 1:**
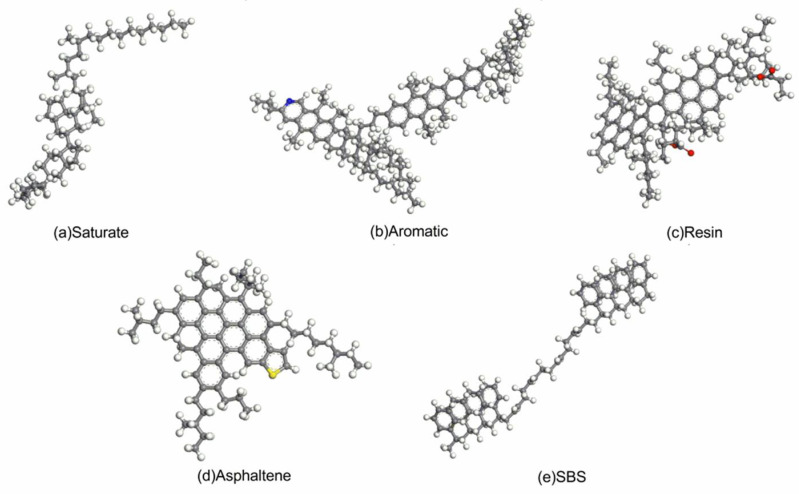
Five initial molecular models: (**a**) saturate; (**b**) aromatic; (**c**) resin; (**d**) asphaltene; (**e**) SBS.

According to the four-component method of asphalt, asphalt can be divided into the saturate, aromatic, resin, and asphaltene [17]. Because asphalt was extremely complex [18,19] and the molecular weight of a single molecule was large, it was not easy to calculate using real asphalt molecule; therefore, the components of asphalt were simplified. The saturate molecule was composed of branched straight-chain alkanes and cycloalkanes [19,20,21,22], the ratio of hydrogen atoms to carbon atoms was about 2 [23], and the simplest molecular formula contained about 50 carbon atoms [24,25]. The aromatic molecule was composed of low-molecular-weight naphthenic aromatic compounds in asphalt, and the ratio of hydrogen atoms to carbon atoms was about 1.5 [20,23,25]. The resin molecule was composed of aromatic rings with a few short side chains and naphthenes [23,25,26], at the same time, all the oxygen bound to carbon on the asphalt constitute hydroxyl, carboxyl, carbonyl, and ester groups, of which ester groups account for the vast majority of these oxygen-containing functional groups. The asphaltene molecule contained fused aromatic rings [15], the most likely structure was 4–10 fused rings, the ratio of hydrogen atoms to carbon atoms was between 0.98 and 1.56 [23], the largest molecular weight [27] contains sulfur, nitrogen, and oxygen elements [28]. According to the typical basic structural units of each component of asphalt currently studied and the typical characteristics, the saturate, aromatic, resin and asphaltene molecules were built. As shown in Figure 1a–d, the molecular formulas of the saturate, aromatic, resin and asphaltene molecules are $C_{51}H_{92}$ $C_{103}H_{151}N$, $C_{95}H_{120}O_{4}$, and $C_{66}H_{80}S$, respectively. 

SBS is a triblock copolymer composed of PS and PB [12]. Because the actual molecular weight was too large, the calculation was beyond the scope of computing power, and the focus of this article was to study the interface interaction and the reaction between SBS and asphalt, so SBS was simplified. A linear SBS molecule with an S/B ratio of 4:4 was selected as the polymer modifier. The structure of the constructed SBS molecule is shown in Figure 1e, and the molecular formula is $C_{80}H_{90}$.

#### 2.1.2. Calculation Methods

All calculations were performed using DMol3 of Materials Studio, using the generalized gradient approximation (GGA) exchange correlation function, using the basis set of double numerical plus polarization atomic orbitals, and the energy tolerance accuracy was set to 1.0 × 10^−5^ Ha.

**Figure 2:**
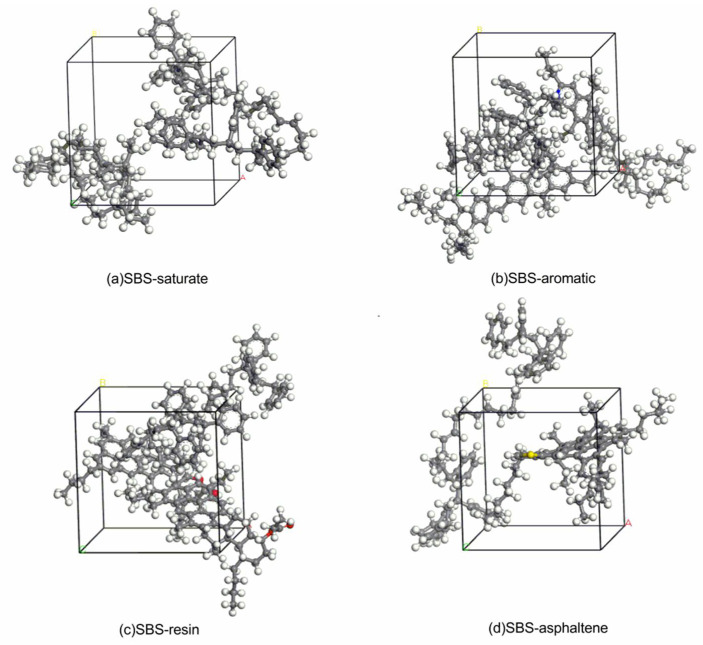
Four system models after energy minimization: (**a**) SBS–saturate; (**b**) SBS–aromatic; (**c**) SBS–resin; (**d**) SBS–asphaltene.

Respectively optimized, the geometrical structure of the SBS, saturate, aromatic, resin, and asphaltene molecules. After the optimization was completed, the SBS molecule was added to the saturate, aromatic, resin and asphaltene molecules to form systems of SBS and asphalt component molecules. The geometric structure of these systems were optimized again to minimize the energy of these systems. Figure 2a–d are the reasonable stable structure after energy minimization of the SBS–saturate system, SBS–aromatic system, SBS–resin system, and SBS–asphaltene system, respectively.

### 2.2. Experimental Materials and Methods

First, measured the FTIR of SBS and matrix asphalt, then used a high shear mixer to prepare SBS modified asphalt. The matrix asphalt was heated to about 150 °C, then SBS was added to the matrix asphalt by using a high-speed shear mixer (6000 rpm, IKA, Regierungsbezirk Freiburg, BW, Germany), and shear stirred for 30 min to obtain SBS modified asphalt. The 791H SBS and 70A asphalt used in this study were produced by Hunan Yueyang Baling Petrochemical Co., Ltd. (Yueyang, China) and Hunan Baoli Asphalt Co., Ltd. (Changsha, China), respectively.

## 3. Results and Discussion

### 3.1. Vibration Spectrums Analysis

The molecular vibration frequency can reflect the molecular structure. Different chemical bonds have different vibration frequencies. The abscissa of molecular vibration spectrum represents the vibration frequency of the chemical bond, and the ordinate represents the vibration intensity of the chemical bond. The vibrations of different chemical bonds have different positions on the abscissa of the peaks formed on the spectrum. In other words, by observing the position of the peaks on the abscissa of the vibrational spectrum, the chemical bonds contained in the material can be judged and the group structure existing in the material can be inferred. If a new peak appears on the abscissa of the vibrational spectrum at a new position, it means that a new chemical bond is formed after the reaction, which proves that a chemical reaction has occurred between the two molecules. Otherwise, it means that no chemical reaction has occurred. In this study, by comparing and analyzing vibration spectrums, it is inferred whether there are bonds breakage and generation between the SBS and four components of asphalt, whether chemical reactions occurred. In this way, the interaction between SBS and asphalt can be judged, the modification mechnism of SBS modified asphalt can be further determined.

#### 3.1.1. SBS–Saturate System Vibration Spectrums

**Figure 3:**
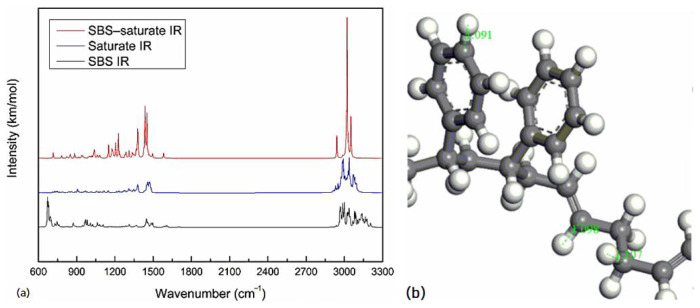
Molecular vibrational spectrums and bond length: (**a**) SBS IR, saturate IR, SBS–saturate IR; (**b**) C–H bond length of SBS.

The molecular vibration spectrums of the SBS molecule, saturate molecule, and SBS–saturate system after the geometric structure optimization are shown in Figure 3a. Assignations of the bands of the SBS molecule vibration spectrum is shown in Table 1. SBS IR in Figure 3a, the peak at 671.66 cm^−1^ is very strong, which is caused by the out-of-plane bending vibration of the C–H bond of the benzene ring [7]. The out-of-plane bending vibration frequency of the C–H bond of the benzene ring is between 670 and 860 cm^−1^, the peak appearing in the interval is often the strongest in the spectrum, and the position of the peak depend on the position of the benzene ring substituent [29].The position of the substituent of the benzene ring on the SBS molecule is 0, and the vibration frequency is the lowest, 671.66 cm^−1^ is very close to 670 cm^−1^, which is obviously the out-of-plane bending vibration frequency of the C–H bond of the benzene ring. In Figure 3a, the stretching vibration frequencies of the C–H bond of the aromatic hydrocarbon and olefin are higher than that of the C–H bond of the alkane. This was because the C atoms of alkane and olefin were bonded to the s orbital of H atoms by sp^3^ and sp^2^ hybrid orbitals, respectively, and the degree of track overlap increased by sp^3^ and sp^2^, the more the track overlap, the shorter the bond length, the greater the bond energy, the greater the force constant, the higher the stretching vibration frequency [29]. At the same time, the stretching vibration frequency of the C–H bond of the aromatic hydrocarbon is higher than that of the C–H bond of the olefin. This was due to the π–π conjugate effect, which caused the single bond connected to it slightly shorter, the force constant increased, the stretching vibration frequency moved to high frequency, and the larger the π–π conjugate system, the more significant the π–π conjugate effect [29]. As shown in Figure 3b, the length of the C–H bond of the alkane of the SBS molecule is 1.107 Å, the length of the C–H bond of the olefin is 1.098 Å, and the length of the C–H bond of the benzene ring is 1.091 Å. With the shortening of the bond length, the higher the vibration frequency of C–H bond is.

Assignations of the bands of the saturate molecule vibration spectrum is shown in Table 2. Because the saturate molecule contained five or six membered rings, the rings had certain tension, which caused the stretching vibration frequency of the C–H bond on the rings moved towards high frequency [29]. The five-membered ring is smaller than the six-membered ring, so the tension is stronger, so the vibration frequency is higher.

Compared with the three vibration spectrums in Figure 3a, it can be found that except for the change of the intensity of the ordinate, the peaks of SBS molecule and the saturate molecule still exist on the spectrum of the SBS–saturate system, and there is no new peak. The position of the vibration spectrum abscissa represents the type of different chemical bond, and the intensity in the direction of the ordinate represents the strength of the interaction of different chemical bond. If the chemical reaction occurred between the SBS molecule and the saturate molecule, the vibration spectrum of the SBS–saturate system might disappear at the old position and appear at the new position. However, there is no peak in the about 1600–2900 cm^−1^ region of SBS molecule and saturate molecule, and no peak appear in the about 1600–2900 cm^−1^ region of SBS–saturate system. This shows that there is no breakage of the old bond and the generation of new bond between the SBS molecule and the saturate molecule of asphalt. It shows that there is no chemical reaction between the SBS molecule and the saturate molecule of asphalt, they are physical blending. In the SBS–saturate system, the intensity of the peaks is significantly increased. This was because the SBS molecule was added to the saturate molecule. There was a physical interaction between the two molecules, which produced molecular resonance, resulting in enhancing in the strength between the bonds.

#### 3.1.2. SBS–Aromatic System Vibration Spectrums

**Figure 4:**
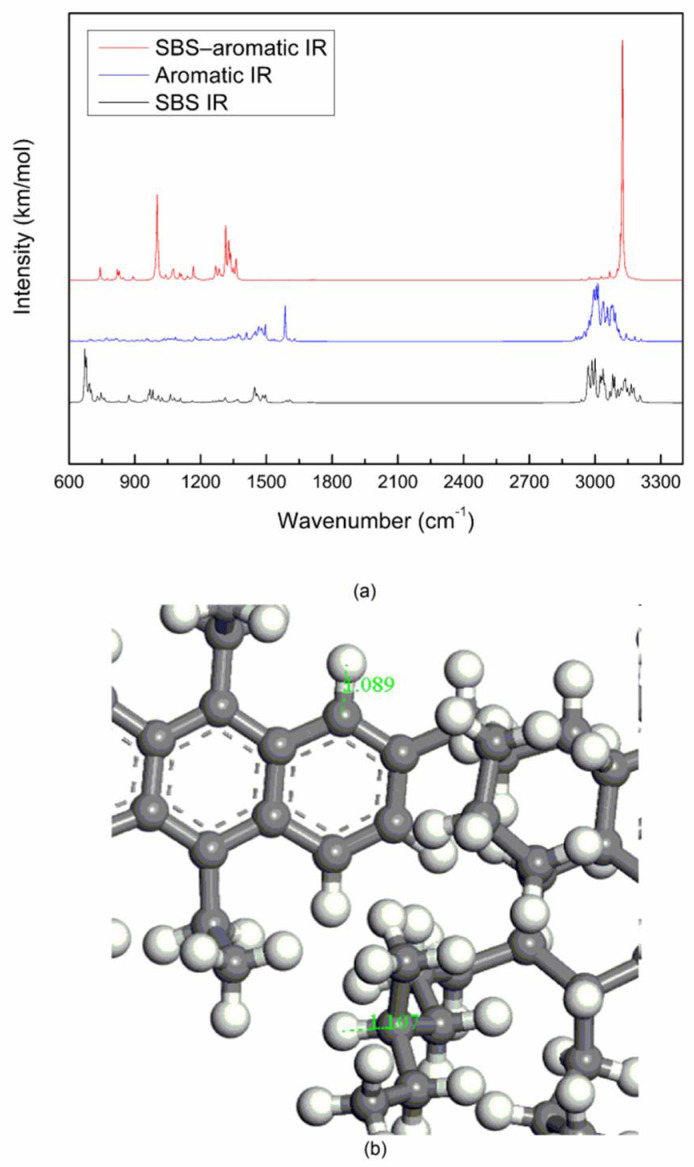
Molecular vibrational spectrums and bond length: (**a**) SBS IR, aromatic IR, SBS–aromatic IR; (**b**) C–H bond length of aromatic.

After optimizing the geometric structure of the SBS molecule, the aromatic molecule and the SBS–aromatic system, the calculated molecular vibration spectrums are shown in Figure 4a. Assignations of the bands of the aromatic molecule vibration spectrum is shown in Table 3. The stretching vibration frequency of the C=N bond is between 1640 and 1680 cm^−1^, and the stretching vibration frequency of the C=C bond of the aromatic ring is between 1370 and 1610 cm^−1^ [29]. Aromatic IR in Figure 4a, the peak at 1585.85 cm^−1^ is caused by the stretching vibration of the C=N bond. Because the nitrogen atom was connected to 5 carbon atoms, the benzene-like six-membered fused aromatic heterocycle was formed, which caused the stretching vibration frequency of the C=N bond to move to a low frequency. As shown in Figure 4b, the length of the C–H bond of the alkane of the aromatic molecule is 1.107 Å, and the length of the C–H bond of the benzene ring is 1.089 Å. The shorter the bond length, the higher the stretching vibration frequency of the C–H bond.

Comparative analysis of Figure 4a show that the band of the SBS–aromatic system only has the intensity change of the ordinate, and the peaks of SBS and aromatic still exist on the band of the SBS–aromatic system, there is no peak at other new position. There is no peak in the about 1700–2900 cm^−1^ region of SBS molecule and aromatic molecule, and no peak in the about 1700–2900 cm^−1^ region of SBS–aromatic system, which shows that there is no breakage of old bond and the formation of new bond between SBS molecule and the aromatic molecule of asphalt, they are mainly physical blending.

#### 3.1.3. SBS–Resin System Vibration Spectrums

**Figure 5:**
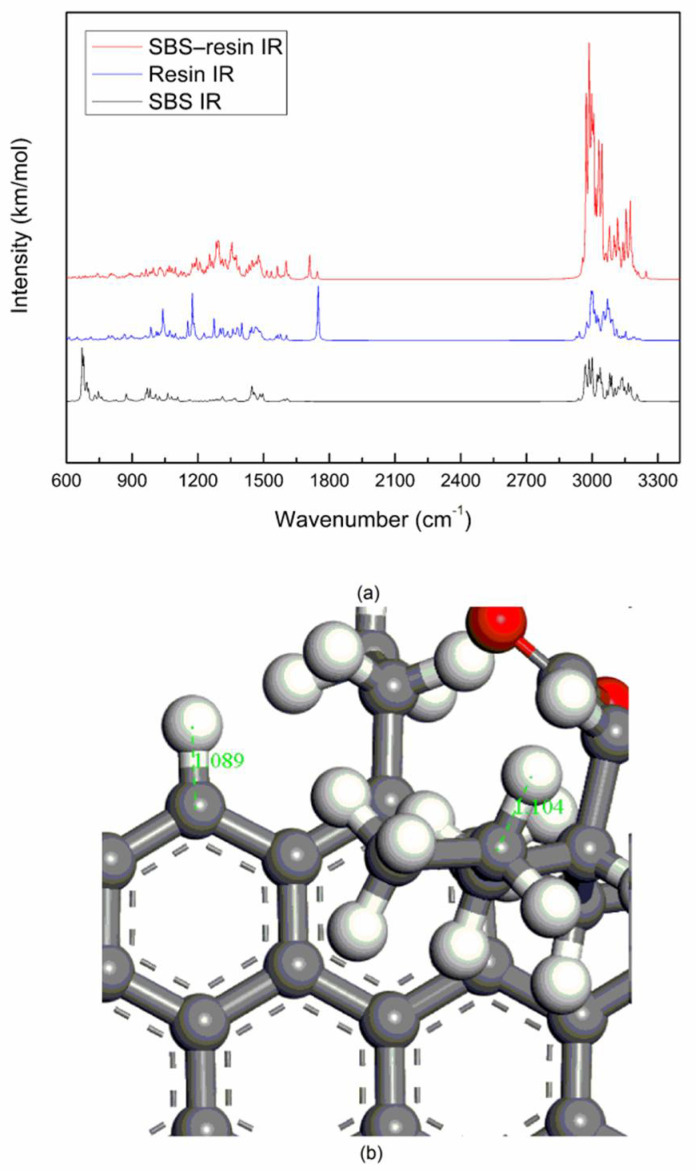
Molecular vibrational spectrums and bond length: (**a**) SBS IR, resin IR, SBS–resin IR; (**b**) C–H bond length of resin.

Figure 5a are the calculated molecular vibration spectrums of the SBS molecule, the resin molecule and the SBS–resin system after the geometric structure are optimized. Assignations of the bands of the resin molecule vibration spectrum is shown in Table 4. The symmetric stretching vibration peaks of the C–O–C bond of the ester group are located at 1000–1050 cm^−1^, and the anti-symmetric stretching vibration peaks are located at 1160–1240 cm^−1^ [29]. Resin IR in Figure 5a, the peaks at 1039.89 cm^−1^ and 1174.91 cm^−1^ are caused by the symmetric stretching vibration and anti-symmetric stretching vibration of the C–O–C bond of the ester group, respectively. The peak at 1749.92 cm^−1^ is caused by the stretching vibration of the C=O bond of the ester [20], which is the strongest peak in the vibrational spectrum of the resin molecule. As shown in Figure 5b, the length of the C–H bond of the alkane of the resin molecule is 1.104 Å, and the length of C–H bond of benzene ring is 1.089 Å, so the vibration intensity of the C–H bond of benzene ring is stronger than that of the alkane.

Comparing Figure 5a, it can be found that the peaks of SBS molecule and the resin molecule still exist on the band of the SBS–resin system, and no peak at new position appear. There is no peak in the about 1800–2900 cm^−1^ area of the SBS–resin system, which shows that there is no breakage of the old bond and the generation of new bond between the SBS molecule and the resin molecule of asphalt, they are also physical blending.

#### 3.1.4. SBS–Asphaltene System Vibration Spectrums

**Figure 6:**
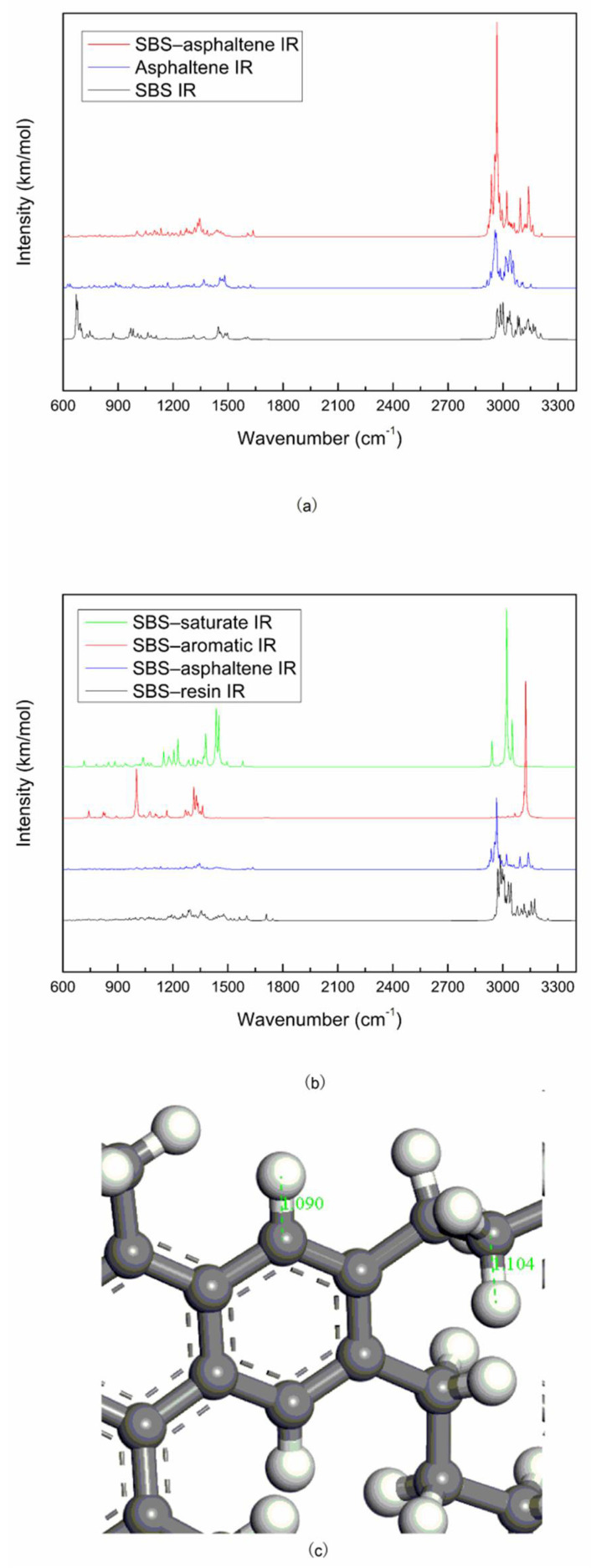
Molecular vibrational spectrums and bond length: (**a**) SBS IR, asphaltene IR, SBS–asphaltene IR; (**b**) SBS–resin IR, SBS–asphaltene IR, SBS–aromatic IR, SBS–saturate IR; (**c**) C–H bond length of asphaltene.

The molecular vibration spectrums of the optimized SBS, asphaltene and the SBS–asphaltene system are shown in Figure 6a. Assignations of the bands of the asphaltene molecule vibration spectrum is shown in Table 5. Asphaltene IR in Figure 6a, the peak at 1170.59 cm^−1^ is caused by the stretching vibration of the C=S bond, and the stretching vibration peak of the C=S bond is located at 1020–1200 cm^−1^ [29]. The sulfur atom was connected to 4 carbon atoms to form a five-membered fused aromatic heterocycle. Due to the π–π conjugation effect, the double bond was slightly elongated, the force constant was reduced, and the frequency of the stretching vibration peak moved to a low frequency [29]. In Figure 6c, the length of the C–H bond of the alkane of the resin molecule is 1.104 Å, and the length of the C–H bond of the benzene ring is 1.090 Å, the vibration intensity of the C–H bond of the benzene ring is stronger than that of the alkane.

Compared with Figure 6a, it can be found that the SBS–asphaltene system has no new peak except for the change of the strength of the ordinate, and there is no new peak in the about 1700–2900 cm^−1^ area of the SBS–asphaltene system. This shows that SBS molecule and the asphaltene molecule of asphalt are still physical blending.

In the analysis of Figure 6b, the strengths of the ordinate of SBS–saturate system and SBS–aromatic system are similar, the strengths of the ordinate of SBS–resin system and SBS–asphaltene system are similar, and the strengths of the ordinate of the vibration spectrums of the first two systems are significantly stronger than those of the latter two systems. Studies had shown that from the saturate, aromatic, resin to asphaltene of asphalt, its molecular weight, aromatic content, and polarity gradually increased [23]. The solubility parameter of the PB blocks were similar to those of the saturate, the solubility parameters of the PS blocks were similar with aromatic, the PB blocks were swollen by the saturate while the PS blocks were swollen by the aromatic [30]. When SBS was added to the asphalt, the PB blocks interacted with the light components of the asphalt (saturate and aromatic), and the PS blocks interacted with the heavy components of the asphalt (resin and asphaltene). Masson et al. [31] had pointed out that the intermolecular interaction between asphalt and PB was stronger than that between asphalt and PS. Therefore, the intensity of the vibration spectrum of SBS and the light components of asphalt is stronger than that of SBS and the heavy components of asphalt.

### 3.2. FTIR Analysis

**Figure 7:**
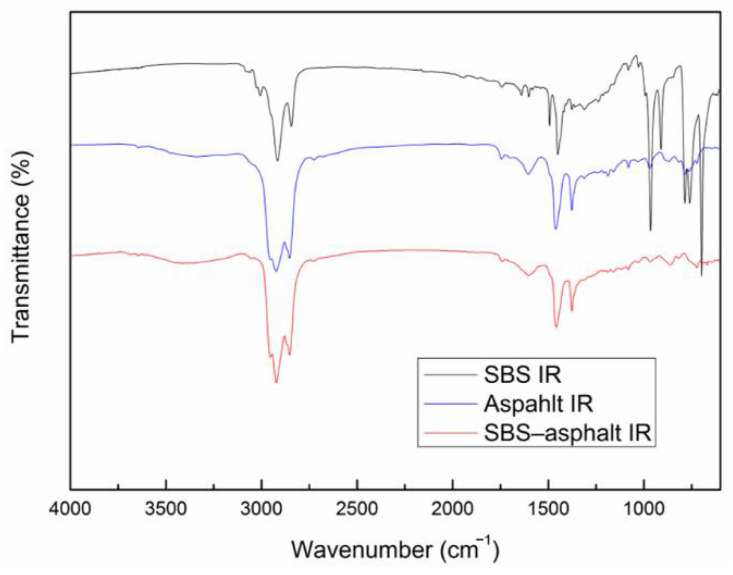
FTIR of SBS, asphalt, and SBS–asphalt.

FTIR can detect the types of the chemical bonds contained in the material. Because for FTIR testing, a given type of bond can only absorb energy corresponding exactly to a specific infrared frequency [18]. Therefore, the characteristic bands in the absorption spectrum of the transmitted IR radiation can be used to identify the bond types to obtain material information about chemical bonds and material structures [3]. If a new band appears on the abscissa of FTIR at a new position, it means that a new chemical bond is formed after the reaction, which proves that there is a chemical reaction. Otherwise, it means that no chemical reaction has occurred. The FTIR of SBS, asphalt and the prepared SBS–asphalt composite are shown in Figure 7, respectively. Assignations of the bands of the SBS FTIR is shown in Table 6. The peak at 697.7 cm^−1^ and the peak at 671.66 cm^−1^ of the SBS IR in Figure 3a have the same meaning. Both peaks are caused by the out-of-plane bending vibration of the C–H bond of the benzene ring [7], which are characteristic peaks of SBS. Asphalt IR in Figure 7, the peaks in the region of 2697.93–3127.97 cm^−1^ are caused by the stretching vibration of the C–H bond.

Comparing with Figure 7, it can be found that in the area of about 1800–2700 cm^−1^, there is no new peak in FTIR of SBS–asphalt, indicating that there is no fracture and formation of chemical bond between SBS and asphalt, and no chemical reaction occurs in the system. SBS modified asphalt is indeed a physical modification.

Both molecular vibrational spectrums and FTIR indicate that SBS and asphalt are physical modification. Grafting SBS and using it as the asphalt modifier is an effective way to increase the chemical effect of SBS modified asphalt. Ortega et al. [32] found that there was a chemical reaction between the anhydride group of dodecenyl succinic anhydride (DSA) and asphalt. By grafting the rubber with acrylamide and then modifying the asphalt, Xie et al. [33] found that chemical interactions between the basic amide group of acrylamide and asphalt occurred. Therefore, the graft activation of SBS can cause the asphalt to react with the grafted part, which can increase the chemical effect between SBS and asphalt.

### 3.3. Binding Energy

A system composed of two or more components, and there is mutual attraction between the components to bind them together. If the components are separated to "infinity", certain amount of energy is needed to overcome their mutual attraction. The amount of energy required indicates how tightly each component is combined, which is called the binding energy of the system. The greater the negative value of the binding energy, the tighter the combination of the system and the more stable the system. Because it means that more energy needs to be invested to separate the entire system. For this article, ΔE_b_ is the energy change when each component molecule of asphalt and SBS molecule are combined. The binding energy ΔE_b_ between each component molecule of asphalt and SBS molecule is as follows:

ΔE_b_ = ΔE_asphalt component/SBS_ − ΔE_asphalt component_ − ΔE_SBS_ (1)

In the formula, the parameter ΔE_asphalt component/SBS_ represents the total energy of the system after adding SBS molecule. ΔE_asphalt component_ and ΔE_SBS_ are the total energy of each component molecule of asphalt and SBS molecule, respectively. The energy of the optimized structure of SBS and each component molecule of asphalt is lower than the sum of the energy of the two molecules that make up the system. This lower energy means that the system composed of SBS and each component molecule of asphalt released energy in the process of structural optimization. The ΔE_b_ values between the saturate molecule, aromatic molecule, resin molecule, asphaltene molecule and SBS molecule are shown in Table 7.

The ΔE_b_ values of the SBS–saturate system, SBS–aromatic system, SBS–resin system, and SBS–asphaltene system are all less than zero, so SBS can be combined with asphalt spontaneously in theory. From the point of view of the binding energy value, the absolute value are not large, indicating that under normal circumstance, the binding between the molecules of SBS and each component of asphalt is not particularly stable, and the system may only have physical adsorption.

In addition, the ΔE_b_ of the SBS–saturate system is the smallest, which indicates that the combination of SBS molecule and the saturate molecule has the worst stability, while the absolute value of binding energy between the aromatic, resin, asphaltene molecules, and SBS molecule are all greater than that between the saturate molecule and SBS molecule. This indicates that SBS is mainly combined with molecules containing aromatic hydrocarbon structure in the asphalt to achieve a uniform mixing state. The possible reason was that the benzene ring in SBS easily interacted with the aromatic, resin, and asphaltene to generate aromatic ring accumulation, thereby achieving a more stable binding state than the saturate. Dong et al. [12] had found that the content of aromatic hydrocarbon in asphalt played an important role in the compatibility of asphalt and polymer. The higher the content of aromatic hydrocarbon, the better the performance of modified asphalt. In asphalt, the aromatic, resin, and asphaltene all contain a large amount of benzene rings, so the combination is more stable, which is consistent with the research results of this article. Therefore, adding aromatic oils can improve the compatibility between SBS and asphalt with the low aromatic hydrocarbon content [31]. However, it should be noted that high aromatic hydrocarbon content will have a bad influence on the performance of modified asphalt after aging. Zhang et al. [8] modified Alfa-70 and Poly-90 asphalts with SBS, and their saturates content was 23.36% and 16.75%, respectively. After the aging test, they found that Poly-90 asphalt was more severely affected.

## 4. Conclusions

Based on the first-principles, this paper analyzed the molecular vibration spectrums of the linear SBS molecule with an S/B ratio of 4:4, saturate molecule, aromatic molecule, resin molecule, asphaltene molecule, SBS–saturate system, SBS–aromatic system, SBS–resin system, and SBS–asphaltene system, and analyzed the interaction between SBS and each component of asphalt from the molecular level. At the same time, the binding strength between SBS and each component of asphalt was analyzed by binding energy. The main conclusions are as follows:

(1) The spectrum of the mixed system of SBS and asphalt is mostly the superposition of the spectrum of SBS and asphalt. The peak position of the mixed system does not change significantly, and the physical effect is mainly between SBS and asphalt.
(2) The vibrational peak intensity of SBS and the light components of asphalt (saturate and aromatic) is stronger than that of SBS and the heavy components of asphalt (resin and asphaltene), which may be due to the interaction of PB blocks and the light components of asphalt, the interaction of PS blocks and the heavy components of asphalt, while the molecular interaction of the former is stronger than that of the latter.
(3) SBS and each component of asphalt can be combined spontaneously. From the absolute value of the binding energy between SBS and each component of asphalt, the stability of the binding between SBS and the saturate is the worst. SBS mainly produces physical adsorption with molecules containing aromatic ring structure in asphalt to achieve a uniform mixing state. This adsorption may be the interaction of aromatic ring packing.

## Figures and Tables

**Table 1:** Assignations of the bands of the SBS molecule vibration spectrum.

Wavenumber (cm^−1^)	Assignations
671.66	δ C–H of the benzene ring (out-of-plane bending)
2937.23–3011.49	ν C–H of the alkane
3011.49–3058.04	ν C–H of the olefin
3058.04–3208.44	ν C–H of the benzene ring

ν = stretching; δ = bending.

**Table 2:** Assignations of the bands of the saturate molecule vibration spectrum.

Wavenumber (cm^−1^)	Assignations
2910.92–3063.11	ν C–H of the alkane
3063.11–3086.71	ν C–H of the six-membered ring
3086.71–3094.97	ν C–H of the five-membered ring

ν = stretching.

**Table 3:** Assignations of the bands of the aromatic molecule vibration spectrum.

Wavenumber (cm^−1^)	Assignations
1585.85	ν C=N
2898.92–3023.91	ν C–H of the alkane
3023.91–3209.58	ν C–H of the benzene ring

ν = stretching.

**Table 4:** Assignations of the bands of the resin molecule vibration spectrum.

Wavenumber (cm^−1^)	Assignations
1039.89	ν s C–O–C of the ester group
1174.91	ν as C–O–C of the ester group
1749.92	ν C=O of the ester
2927.34–3038.96	ν C–H of the alkane
3038.96–3218.92	ν C–H of the benzene ring

ν = stretching; s = symmetric; as = asymmetric.

**Table 5:** Assignations of the bands of the asphaltene molecule vibration spectrum.

Wavenumber (cm^−1^)	Assignations
1170.59	ν C=S
2927.34–2999.7	ν C–H of the alkane
2999.7–3218.92	ν C–H of the benzene ring

ν = stretching.

**Table 6:** Assignations of the bands of the SBS FTIR.

Wavenumber (cm^−1^)	Assignations
2814.12–2990.57	ν C–H of the alkane
2990.57–3049.39	ν C–H of the olefin
3049.39–3117.37	ν C–H of the benzene ring

ν = stretching.

**Table 7:** The binding energy between each component molecule of asphalt and SBS molecule.

Binding Energy	Saturate	Aromatic	Resin	Asphaltene
$E_{b}\left(\mathrm{Ha}\right)$	−0.3139	−0.3809	−0.3663	−0.3805

## Data Availability

Data is contained within the article.

## Abbreviations

SBS	Styrene–butadiene–styrene
DFT	Density functional theory
FTIR	Fourier transform infrared spectroscopy
PB	Polybutadiene
PS	Polystyrene
SBR	Styrene–butadiene rubber
EVA	Ethylene–vinyl acetate
UV	Ultraviolet
MD	Molecular dynamics
GGA	Generalized gradient approximation
DSA	Dodecenyl succinic anhydride
